# Mutations in blind cavefish target the light-regulated circadian clock gene, *period 2*

**DOI:** 10.1038/s41598-018-27080-2

**Published:** 2018-06-08

**Authors:** Rosa Maria Ceinos, Elena Frigato, Cristina Pagano, Nadine Fröhlich, Pietro Negrini, Nicola Cavallari, Daniela Vallone, Silvia Fuselli, Cristiano Bertolucci, Nicholas S. Foulkes

**Affiliations:** 1 Institute of Toxicology and Genetics, Karlsruhe Institute of Technology, Eggenstein-Leopoldshafen, Germany; 2 Department of Life Sciences and Biotechnology, University of Ferrara, Ferrara, Italy; 3 Present Address: Facultade de Bioloxía, Universidade de Vigo, Vigo, Spain; 4 Present Address: Institute of Science and Technology, Klosterneuburg, Austria; 5 Present Address: CNR, ISASI “E. Caianiello” Pozzuoli, Naples, Italy

## Abstract

Light represents the principal signal driving circadian clock entrainment. However, how light influences the evolution of the clock remains poorly understood. The cavefish *Phreatichthys andruzzii* represents a fascinating model to explore how evolution under extreme aphotic conditions shapes the circadian clock, since in this species the clock is unresponsive to light. We have previously demonstrated that loss-of-function mutations targeting non-visual opsins contribute in part to this blind clock phenotype. Here, we have compared orthologs of two core clock genes that play a key role in photic entrainment, *cry1a* and *per2*, in both zebrafish and *P. andruzzii*. We encountered aberrantly spliced variants for the *P. andruzzii per2* transcript. The most abundant transcript encodes a truncated protein lacking the C-terminal Cry binding domain and incorporating an intronic, transposon-derived coding sequence. We demonstrate that the transposon insertion leads to a predominantly cytoplasmic localization of the cavefish Per2 protein in contrast to the zebrafish ortholog which is distributed in both the nucleus and cytoplasm. Thus, it seems that during evolution in complete darkness, the photic entrainment pathway of the circadian clock has been subject to mutation at multiple levels, extending from opsin photoreceptors to nuclear effectors.

## Introduction

The circadian clock plays a central role in timing most behavioural and physiological functions. By generating an endogenous rhythm with a period of ~24 h, the clock enables organisms to anticipate day-night changes in their environment and thereby enhances their survival. Ambient environmental signals indicative of the time of day (so-called *zeitgebers*, or time givers), primarily light, reset the phase of this clock on a daily basis to ensure it remains synchronised with the 24 hours day-night cycle^1^. In mammals, a central circadian clock located in the suprachiasmatic nucleus (SCN) of the hypothalamus is indirectly synchronised by light perceived by intrinsically photosensitive retinal ganglion cells (ipRGCs) in the retina. Light signals are conveyed from the eye to the SCN clock via the retinohypothalamic tract. The SCN in turn relays timing information to an array of peripheral clocks distributed in most tissues via complex systemic signals^2^. Interestingly, in the case of teleosts, light directly entrains the peripheral clocks in tissues, organs and even cell lines as well as in central pacemaker structures such as the photosensitive pineal gland. Indeed, most cell types express photoreceptors and exhibit directly light inducible gene expression^3^.

At the molecular genetic level, interconnected transcription-translation feedback loops (TTFLs) constitute the core of the circadian clock mechanism. In vertebrates, the bHLH PAS transcription factors Bmal and Clock activate the transcription of *period* (*per*) and *cryptochrome* (*cry*) genes via binding to E-box promoter elements in their promoters. As the levels of Per and Cry proteins rise, they form a complex that is translocated to the nucleus. In the nucleus Per and Cry proteins inhibit the Clock/Bmal-dependent transactivation of the *per* and *cry* genes^4^. The resulting reduction in Per and Cry protein levels ultimately releases Clock and Bmal to drive a new cycle of activation. In parallel, Clock and Bmal together with Cry and Per regulate networks of clock controlled genes via E-box enhancer elements. Transient transcriptional induction of a subset of core clock genes in response to light exposure appears to serve as a key step in entrainment of the clock by the day-night cycle. For example, light induced expression of *per1* and *per2* in the SCN has been extensively studied in the mouse^5,6^. Furthermore, in zebrafish cells the *per2* and *cry1a* genes are strongly induced upon direct exposure to light via the effect of D-box enhancer promoter elements^7,8^. As well as serving to periodically down-regulate Clock/Bmal-dependent transactivation within the core clock mechanism, Per proteins appear to exhibit more complex functions with reports of both positive and negative regulation of other transcription factor systems^9^. Furthermore, the subcellular localization of many clock proteins and, in particular, the timing of their entry into the nucleus represent key properties, ensuring characteristic delays in the progression of the core feedback loop and ensuring that this timing mechanism generates rhythms with a circadian period.

One poorly understood issue is how long-term changes in environmental lighting conditions have shaped the evolution of the clock mechanism. Blind cavefish represent fascinating models to study this issue due to their set of striking adaptations (so-called troglomorphisms), resulting from evolution under perpetual darkness. Most notably, with increasing time isolated in a dark environment, cavefish progressively lose dedicated photoreceptive organs such as the eyes and pineal organ, instead relying on enhancement of non-visual senses to navigate and find food^10,11^. Troglomorphic phenotypes also include loss of body pigment, as well as reduced metabolic rate. One important cavefish model is the Somalian cavefish *Phreatichthys andruzzii*, which exhibits an extreme troglomorphic phenotype as the result of evolution for approximately 2,5 million years in complete isolation from sunlight beneath the desert^12^. Importantly, in a previous study, we have documented the complete loss of light entrainment of the clock in this species as well as loss-of-function mutations in three, non-visual opsin genes^12,13^.

In this report, we have explored the function of the *cry1a* and *per2* clock genes in *P. andruzzii,* which are normally light inducible in sighted fish species such as the zebrafish. Consistent with the general loss of light induced gene expression in this cavefish species, the expression of neither gene is inducible by light^13^. Here, we reveal that while the zebrafish (zf Cry1a) and *P. andruzzii* Cry1a (pa Cry1a) amino acid sequences are highly conserved, the C-terminal region of the predicted *P. andruzzii* Per2 (pa Per2) sequence differs significantly from the zebrafish ortholog (zf Per2) as a result of altered mRNA splicing. Differential splicing of the pa *per2* transcript leads to insertion of transposon-derived sequences and a premature termination codon, as well as an alteration of the subcellular localization of the predicted protein.

## Methods

### Animals

*Phreatichthys andruzzii* was originally collected from the phreatic layer of the Somali desert (1975–1982, Bud-Bud area), in an area that currently cannot be reached easily. They were subsequently maintained and bred at the University of Ferrara, Italy. Although exhibiting high longevity (more than 40 years in captivity), *P. andruzzii* is a slow-growing species with a reduced number of offspring. In the present study we used individuals from the F1 and F2 generations. The fish were kept in darkness at a constant 27 °C except during food administration and aquaria maintenance. Three times per week the fish were fed with frozen chironomid larvae. All experiments using adult zebrafish were performed using standard methods described elsewhere^14^. All husbandry and experimental procedures complied with European Legislation for the Protection of Animals used for Scientific Purposes (Directive 2010/63/EU). Handling procedures and research protocols were approved by the University of Ferrara (Italy) and Karlsruhe Institute of Technology (Germany) Institutional Animal Care and Use Committees as well as by the Italian Ministry of Health (auth. num. 890/2016-PR).

### Cloning Cavefish cDNA and Genomic Sequences

Samples of total RNA from *P. andruzzii* brain, heart and cell cultures were collected with Trizol (Invitrogen) according to the manufacturer’s instructions. Total RNA was quantified spectrophotometrically using BioSpec-nano (Shimadzu) and samples were stored at −80 °C. One microgram of DNase-treated RNA was transcribed into cDNA with Superscript III RT (Invitrogen) according to the manufacturer’s instructions. To complete the pa *per2* mRNA sequences previously obtained by our group (GenBank acc. no. GQ404487.1), we use a “nested and external” PCR approach designing the forward primer according to the previously cloned cavefish sequence and the reverse primer according to the corresponding zebrafish homolog (Ensembl: ENSDARG00000034503). The primers were designed using Primer3 software and are indicated in Table S1. Additional cDNA sequences were subsequently obtained using a 3′ SMART RACE cDNA amplification kit (BD Bioscience) and then the complete coding sequences of the three cavefish *per2* transcript variants were deposited in GenBank (acc. no: per2_tv1, MF535179; per2_tv2, MF535180 and per2_tv3, MF535181). Genomic DNA was extracted from cavefish caudal fins with a standard phenol-chloroform protocol^15^. To establish whether the insertions found in one of the three transcript variants of *per2* were of intronic origin, primers were designed based on cDNA and genomic sequence from the cavefish *per2* gene at positions flanking predicted exon-intron splicing sites (Table S1).

### Gene Expression Analysis

Quantitative RTPCR (qPCR) was performed for *P. andruzzii per2* transcript variants using the pairs of primers shown in Table S1. The SsoFast EvaGreen Supermix (Biorad) was employed for qPCR reactions according to the manufacturer’s recommendations. The efficiency of primers was verified by constructing standard curves for all PCR products investigated. After amplification, a melting curve analysis was performed to confirm the specificity of the amplicons. The relative levels of each sample were calculated by the 2^−ΔΔCT^ method (where CT is the cycle number at which the signal reaches the threshold of detection)^16^. Relative expression levels were normalized to *β-actin*^13^. Each CT value is the mean of three biological replicates and each assay was performed a minimum of three times.

### Serum shock

PAC-2 and CF1 cells were plated in L15 medium in the presence of 10% serum. At 80% confluence, the concentration of serum was reduced to 0.5% by a medium change and the cells were maintained in darkness. After 48 h, the culture medium was replaced with L15 medium supplemented with 20% serum. Samples were subsequently harvested in darkness at different time-points over a 12 hours period following the serum shock.

### Construction of Plasmids

The ORFs of *per2* from zebrafish and cavefish were subcloned into the expression vector pcDNA3.1/myc-HisA vector (Promega). This vector was initially modified by deleting the Neomycin resistance cassette, SV40 promoter and origin (ΔSV40-ΔNEO): (i) To generate zf*per2*(4.2 kb):pcDNA3.1(+)mycHisA-tagΔNeo, the 4.2 kb zf *per2* ORF was amplified using a forward primer containing a Xba1 site (5′-CTTCTAGACATTAAACCCAAGTCCGATG-3′) and a reverse primer incorporating the Stop codon that was modified to generate an in-frame Xba1 site (5′-CAGTTCTAGAGTCTGGACCGGGACAGC-3′). Following amplification and digestion of the PCR product using XbaI, this was then cloned into the XbaI site of the ΔSV40-ΔNEO vector. (ii) To generate cf*per2*(3,8 kb):pcDNA3.1(+)mycHisA-tagΔNeo, the 3,8 kb pa *per2* ORF was cloned into ΔSV40-ΔNEO using a comparable strategy to that mentioned above except that the forward PCR primer carried a KpnI site (5′-GTGATCTGCTGGTACCTTGCTTTACTTGTTC-3′) and the reverse primer carried a mutagenised Stop codon sequence and one EcoRI site (5′-TTAAGAATTCCAATGCAATATAAGATATGTGT-3′). iii) To generate zf*per2*+Tr:pcDNA3.1(+)mycHisA-tagΔNeo: the cavefish transposon, intron-based sequence identified inside the pa *per2* gene, was inserted into the zf*per2*(4,2 kb):pcDNA3.1(+)mycHisA-tagΔNeo construct at the same position that it is located in the pa *per2* wild-type CDS. This was achieved by generating a NheI restriction site in the zf*per2* coding sequence using the primer, 5′-CAGGTCACGTCTGAAGCTAGCCCTGCTGCTCGCTCAT-3′ in the context of the Quickchange site-directed mutagenesis kit (Stratagene). Simultaneously, the transposon sequence (225 bp) was amplified by PCR from cavefish cDNA with primers that generated NheI sites (Fw: 5′-CATCTGAGGCTAGCCGACTGGTCTTC-3′; Rv: 5′-GGAAGCTAGCTCTCTGTGGTGGTACAA-3′) for cloning of the sequence into the mutagenised vector. iv) To generate zf*per2*-Δ:pcDNA3.1(+)mycHisA-tagΔNeo) we initially created a 650 bp deletion at the 3′ end of zf*per2*. For this, the zf*per2*(4,2 kb):pcDNA3.1(+)mycHisA-tagΔNeo plasmid was amplified by PCR using primers designed by Primer3 software, (Fw:5′-TCTAGAGGGCCCTTCGAACAAAAACTCATCTC-3′ and Rv: 5′-GTCACTTGATGAAGAGAGCGCATCGC-3′). Subsequently, the PCR product was ligated into the pcDNA3.1(+)mycHisA-tagΔNeo vector. v) Finally, to generate zf*per2*+Tr-Δ, which contains the zf*per2* ORF fused with the *P. andruzzii* transposon sequence and carries a 650 bp deletion at the 3′ end, we created a deletion in the plasmid zf*per2*+Tr:pcDNA3.1(+)mycHisA-tagΔNeo using the same strategy that was used to obtain zf*per2* (zf*per2*-Δ:pcDNA3.1(+)mycHisA-tagΔNeo). All constructs were transformed into *E.coli* bacteria (TOP10F’, Invitrogen).

### Cell lines and transfection

The zebrafish embryo-derived cell line PAC-2 and cavefish cell line CF1 derived from caudal fin clips of adult *P. andruzzii*^13^, were cultured in Leibovitz’s L15 medium (Gibco) supplemented with 15% of fetal calf serum (Biochrom KG) (PAC-2 cells) or 20% (CF1 cells), 100 units/ml penicillin, 100 μg/ml streptomycin and 50 μg/ml gentamycin (Gibco) at 26 °C. For transfection, 1 × 10^5^ CF1 or PAC-2 cells were seeded onto 12 mm coverslips housed in 24 multi-well plates the day before transfection. Each expression vector (250 ng) or an empty vector control (250 ng) were transfected using Fugene (Promega) and incubated for 24 hrs at 26 °C. The following day, the medium was renewed. Transfected cells were then maintained in darkness for 2 days or 3 days in LD cycles depending on the experiment, before fixing using 4% paraformaldehyde (PFA) for 20 mins at room temperature. The cells were then processed for immunostaining.

### Immunostaining

Transfected cells fixed in 4% PFA were washed 3 times (each wash 5 mins) in 1xPBS, once with PBST (1xPBS containing 0.1% Tween) and then blocked for 1 hr in PBST-BSA (1% bovine serum albumin in PBST). Subsequently, coverslips were incubated with a primary c-myc antibody (9E19, sc-40 Santa Cruz Biotechnologies), at a 1:1000 dilution in PBST-BSA at 4 °C overnight. Cells were then washed and incubated with a second antibody (Goat anti-mouse, Alexa Fluor 546, Invitrogen) in PBST-BSA at 1:1000 for 1 hr in darkness. Nuclear DNA was stained with blue-fluorescent 4′,6-Diamidino-2-phenylindole (DAPI) at 1:10.000 for 30 mins and finally cells were washed 3 times in PBST for 10 mins each wash. Finally, the coverslips were mounted onto a slide using a drop of fluorescent mounting medium (DAKO).

### Image acquisition and processing

Immunostained cells were visualized using a confocal microscope SPE (Leica) with a 40 × oil immersion objective and images were captured with a digital camera using Leica software. Fluorescence from images was quantified with Image J software. Over each picture a region of interest was defined by drawing a boundary around the nucleus and cytoplasm of the cell body to measure fluorescence intensity. Fluorescence intensity from nuclei was divided by the sum of the total fluorescence in nuclei and cytoplasm. In this program the red positive staining was evaluated as a gray scale value. At least 100 PAC-2 cells or 60 CF1 cells were analyzed per transfection. The representative images shown in this manuscript were prepared using Photoshop CS2 software (Adobe Systems Inc.). The subcellular distribution data are presented in box-and-whisker plot diagrams.

### Molecular evolution analysis

The *P. andruzzii* Cry1a and Per2 protein sequences were used as a query in BLAST protein to retrieve homologous sequences. The corresponding nucleotide coding sequences (CDS) were obtained from GenBank (Table S2). *cry1a* and *per2* CDS were aligned considering their protein translation with MAFFT version 7, scoring matrix BLOSUM62^17^. Besides the full-length *per2* transcript variant, the truncated CDS corresponding to transcript variant 2 (pa per2_tv2) was considered in the analyses. From the aligned protein sequences, we identified the distribution of the amino acid substitutions in functional domains predicted using SMART (http://smart.embl-heidelberg.de) and seqNLS (http://mleg.cse.sc.edu/seqNLS/). The putative functional effect of the amino acid changes in these domains presented exclusively by the cavefish lineage (private substitutions) was predicted using the physiochemical distances available in the Grantham amino acid difference matrix^18^ (Table S3). Maximum likelihood trees for both genes were estimated using the program RAxML 8.1.2^19^ and the GTR + gamma substitution model. The topology of these estimated trees and the gap-free alignment of both genes were used to test if *cry1a* and *per2* in the cavefish lineage (foreground branch) evolve at a different relative rate compared to the other lineages of the phylogeny (background branches). Specifically, we estimated the likelihood of different branch-based models available in CODEML module of PAML package^20^. The simplest models assume the same ratio of non-synonymous to synonymous substitutions (ω = dN/dS) for all branches in the phylogeny (“one-ratio” models); in this kind of model ω can be estimated as a free parameter (Model A in Table S4) of fixed at 1.0 (Model B in Table S4), the expected ω value under neutral evolution. A nested model assumes that the branch of interest (in our case the cavefish) has a dN/dS ratio (ωPa) that is different from the background ratio ω0 (“two-ratio models”, Model C in Table S4). Finally, the “free-ratio” model that assumes an independent dN/dS ratio for each branch in the phylogeny was used to calculate branch-specific rate of variation (Model D Table S4). A likelihood ratio test was used for comparing the goodness of fit of models, one of which (the null model) is a special case of the other (the alternative model with more parameters).

**Figure 1:**
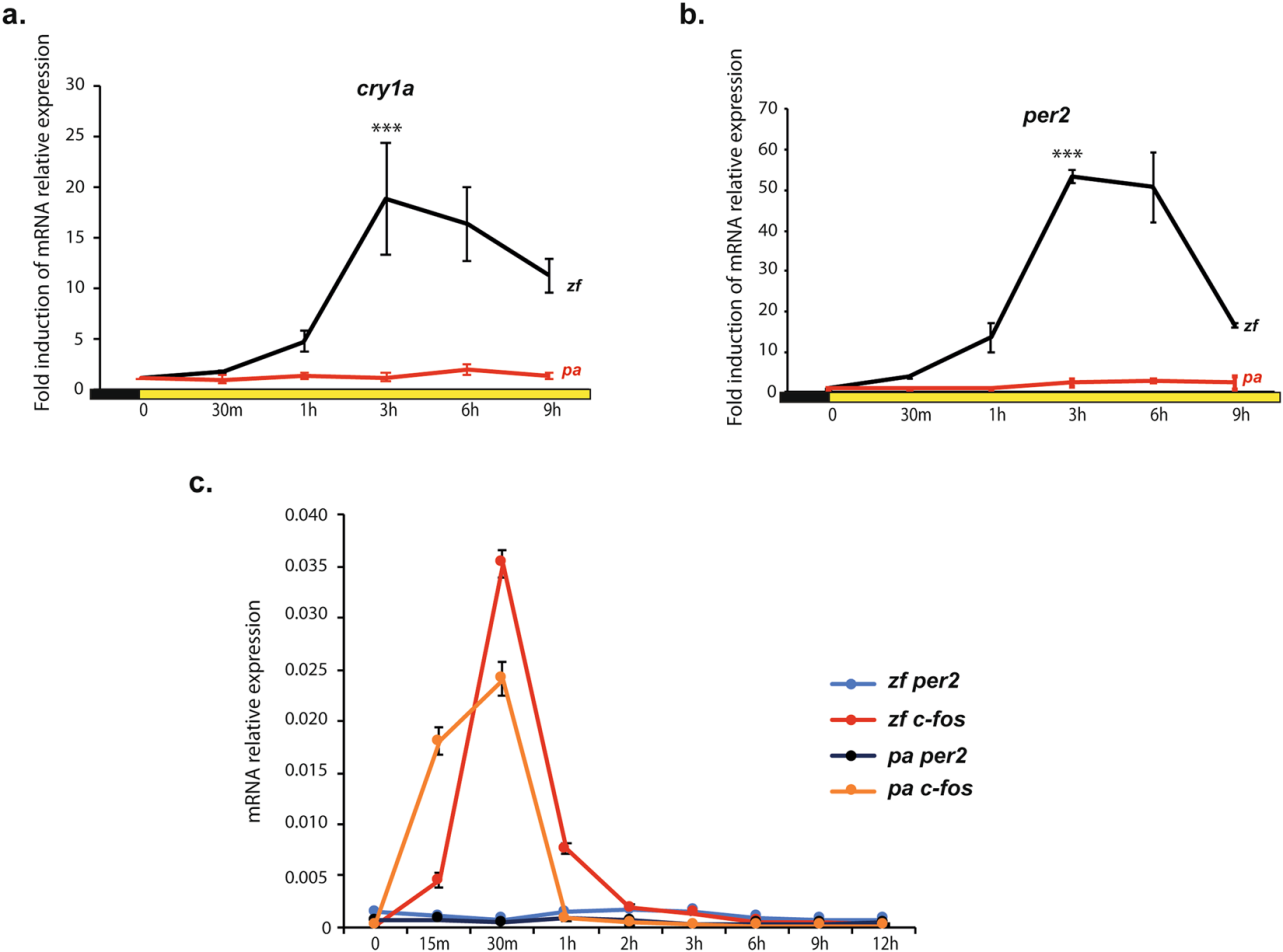
Loss of light induced clock gene expression in *P. andruzzii* CF1 cells. qPCR analysis of *cry1a* (**a**) and *per2* (**b**) from zebrafish (PAC-2) (zf, black traces) and *P. andruzzii* (CF1) (pa, red traces) cell lines. Samples were taken at different time points during 9 hours exposure to light (tungsten light source, 20 μW/cm^2^). Mean fold induction of mRNA relative expression with respect to time 0 (n = 3) ± SD is plotted on the y-axes, whereas time is plotted on the x-axes. Levels of significance between peak points of expression and time 0 are indicated for each gene (***p < 0.001, **p < 0.01, *p < 0.05). Black and yellow bars below the graphics indicate the different lighting conditions during the experiment. Statistical analysis was performed by one-way ANOVA followed by Bonferroni multiple comparison test. (**c**) qPCR analysis of *per2* and *c-fos* mRNA expression from zebrafish (*zf*) and *P. andruzzii* (*pa*) cell lines. Samples were taken at different time points during a 12 hours period following a serum shock. mRNA relative expression with respect to time 0 (n = 3) ± SD is plotted on the y-axis, while time (minutes (m) or hours (h)) is plotted on the x-axis.

### Statistical analyses

Statistical differences were assessed with Kruskal-Wallis followed by Dunn´s multiple comparison test or one-way ANOVA followed by Bonferroni’s multiple comparison test with p < 0.05 considered statistically significant. Statistical analyses were performed in Prism 5.0 (GraphPad Software Inc.) and graphs plotted in Excel (Microsoft). Specific statistical tests applied for each data set are detailed in the corresponding figure legends and the main manuscript text.

## Results

### Mutations in a light inducible clock gene in cavefish

**Figure 2:**
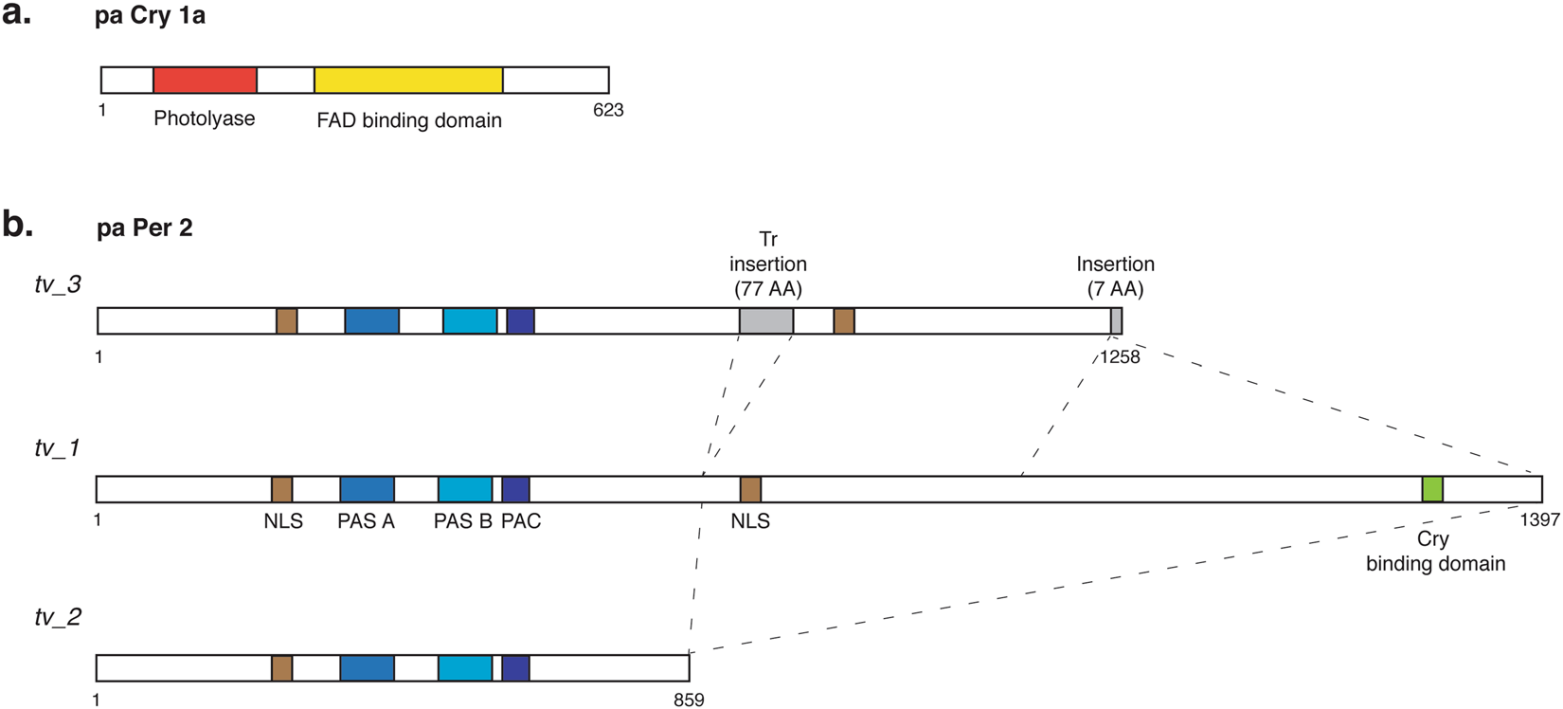
Schematic representation of the predicted proteins encoded by the single pa *cry1a* and three pa *per2* cavefish transcripts. In the case of pa Cry1a (**a**), the red bar represents the Photolyase domain while the yellow bar indicates the extent of the Flavin Adenine Dinucleotide (FAD) binding domain. In the three transcript variants of pa Per2 (**b**), (*tv_*1, *tv_*2 and *tv_*3), brown bars represent nuclear localization signals (NLS), blue bars, the Per, Arnt, Sim (PAS) A domain, light blue bars, the PAS B domain and violet bars, the PAC domain (motif C-terminal to the PAS domains). The green bar shows the position of the Cry interaction domain (absent from isoforms pa Per2_*tv*2 and pa Per2_*tv*3), while the grey bar shows the location of the transposon-derived (Tr) and intronic sequences in pa Per2_*tv*3. Dotted lines indicate the location of amino acid sequences in pa Per2_*tv*2 and pa Per2_*tv*3 relative to pa Per2_*tv*1. Numbers below each protein schematic representation indicate the corresponding amino acid positions.

A key step in photic entrainment of the circadian clock in fish is the acute induction of the *cry1a* and *per2* genes by light. In our previous study, we reported that the expression of both these genes in *P. andruzzii* shows arrhythmic expression upon exposure to a light-dark cycle^13^, compared to the robust oscillation observed with the zebrafish orthologs. Here, we confirm the lack of light responsiveness of these clock genes upon acute exposure to light in the *P. andruzzii* CF1 cell line compared with the robust light driven induction observed in zebrafish PAC-2 cells (Fig. 1a,b and Table S1). Previously, we have demonstrated that serum shock treatment serves as an efficient zeitgeber for the cavefish clock in CF1 cells^13^. We therefore tested whether, as in the case of mammals, *per2* expression may be transiently induced upon serum treatment in cavefish cells^21^. Interestingly, the expression of *per2* is not induced by serum shock treatment either in zebrafish or in the blind cavefish species (Fig. 1c), pointing to a more specialized function for *per2* linked with light responsiveness in fish. A key question is whether loss of light regulated expression represents the only abnormality affecting these two clock genes in the blind cavefish or alternatively if additional mutations might have targeted *cry1a* and *per2* during evolution under constant darkness. We have therefore also scrutinized the coding sequences of these two genes in *P. andruzzii*. In the case of *cry1a*, we identified a single transcript with an open reading frame encoding a 623 amino acids protein (Fig. 2a) that shows high similarity (about 93%) over its entire length with the zebrafish Cry1a ortholog (see also^13^). In particular, the key functional domains of the protein predicted by *in silico* analysis, namely the DNA Photolyase domain, (amino acids 5–170) and the FAD binding domain, (amino acids 213–486), showed high levels of conservation (98% and 96%, respectively).

**Figure 3:**
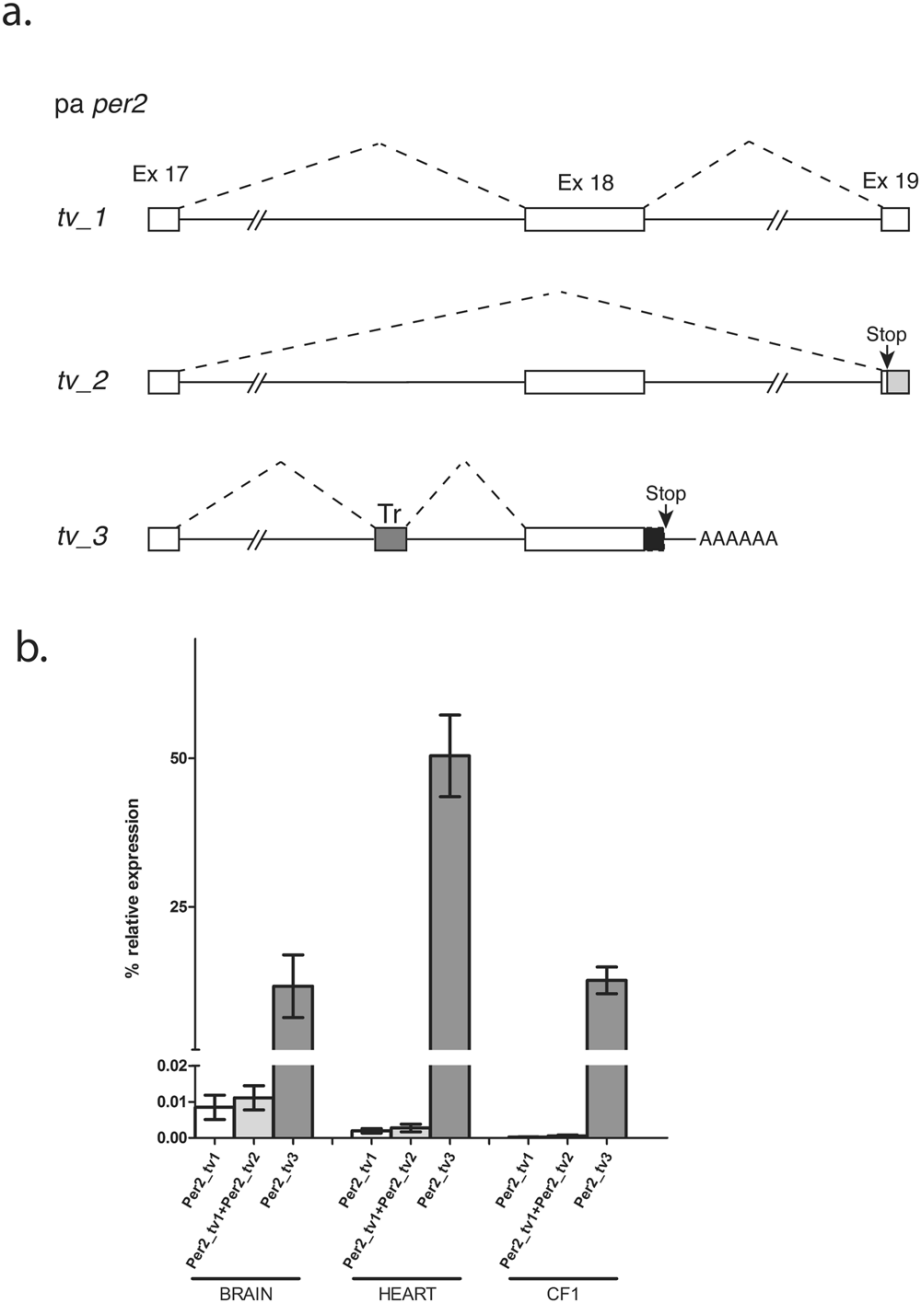
Transcript variants of the *P. andruzzii per2* gene. (**a**) Schematic representation of the portion of the cavefish *per2* gene, between exon 17 and exon 19, where alternative splicing and transcript processing generates the three mRNA transcript variants (*tv_*1, *tv_*2 and *tv_*3). Open boxes denote the coding exons 17, 18 and 19 while a filled dark grey box (Tr) shows the location of transposon-derived sequences which are inserted by aberrant splicing into the *per2* mRNA in transcript variant *tv_*3. “Stop” indicates the premature termination codon and “AAAAAA” the site of premature polyadenylation in the transcript variant *tv_*3. The filled light grey box, present in transcript variant *tv_2*, shows the 3′ untranslated sequence generated in exon 19 by a frame shift caused by the removal of exon 18. Dotted lines indicate the positions of alternative splicing in the three transcripts. (**b**) qPCR analysis of the relative expression levels of the three different *per2* transcript variants in total RNA prepared from cavefish brain, heart and the fin clip-derived cavefish cell line, CF1. Primers used are reported in Table S1. Statistical analysis was performed by Kruskal-Wallis test.

In the case of the pa *per2* gene, we previously cloned only a partial cDNA sequence^13^. In order to characterize this gene further, we decided to use a RTPCR-based cloning strategy to amplify full-length cDNA sequences for the cavefish *per2* gene from adult brain RNA. We identified a set of alternatively spliced transcripts with predicted open reading frames differing at their C-termini (Fig. 2b). One transcript (transcript variant 1, *tv1*) encodes a 1,397 amino acids protein showing a high level of similarity (approximately 89%) with the zebrafish Per2 ortholog (ENSDARG00000034503). This pa *per2_tv1* isoform encompasses all the domains characteristically associated with the Per proteins, namely PAS (PER-ARNt-SIM) A (amino acids 246–313), PAS B (amino acids 386–452), PAC (PAS-associated C-terminal motif, amino acids 459–502) and nuclear localization signals (NLS, amino acids 191–197 and 877–891). However, two additional transcript variants, *tv2* and *tv3*, are predicted to encode smaller proteins of 859 and 1,258 amino acids respectively, which both show C-terminal truncations resulting in the loss of the predicted Cry interaction domain. To explore how these pa *per2* transcript variants are generated, we cloned a subsection of the *P. andruzzii* genomic *per2* locus encompassing the 3′ regions of the transcript variants and by comparison with the zebrafish genome sequence, we determined the exon-intron structure of the cavefish gene. Comparison of this genomic structure with the cDNA sequences of the transcript variants revealed that while pa *per2_tv1* results from the correct splicing of all 3′coding exons (Ex 17–19), in pa *per2_tv2*, exon 18 is excluded (Fig. 3a). This exon skipping results in a frameshift of the coding sequence with the consequent generation of a premature termination codon (Stop) and downstream 3′ untranslated sequence (light grey box) in exon 19. Finally, in the case of the transcript variant *tv3*, the alternative use of donor and acceptor splice sites flanking exon 18 results in the insertion of 225 bp from intron 17 (dark grey box, Tr) upstream, and 25 bp (black box) from intron 18 downstream of exon 18. Importantly, the insertion of the 225 bp intronic sequence does not disrupt the *per2* protein-coding open reading frame and in particular, incorporates a novel region of homology with teleost transposon-encoded reverse transcriptase (dark grey box, Tr) (*Danio rerio*: 72%, acc. no. BAE46430.1; *Anguilla japonica*: 56%, BAD72127.1; *Salmon salar*: 55%, ABQ01988.1). In addition, downstream of exon 18 the inserted 25 bp intron-derived sequence generates a premature termination codon (Stop).

We then investigated the relative levels and patterns of expression of the three transcript variants, performing qPCR analysis of RNA extracts from adult *P. andruzzii* brain, heart and a cell line derived from *P. andruzzii* caudal fin (CF1^13^) (Fig. 3b). Interestingly, while pa *per2_tv1* and _*tv2* show relatively low basal levels in all samples analysed, the pa *per2_tv3* variant shows 2–3 orders of magnitude higher levels of expression in all samples (p < 0.001 for both tissues and the CF1 cell line, Kruskal-Wallis one-way ANOVA), suggesting that a truncated protein lacking the Cry binding domain and including a novel transposon-derived coding sequence represents the most abundant *per2* transcript variant in *P. andruzzii*.

### Molecular evolution of *P. andruzzii* Cry1a and Per2

We next investigated the rate of evolution of the *P. andruzzii cry1a* and *per2* gene sequences in order to gain insight into the selective pressures acting upon these two clock genes in this specific lineage. The alignment of 17 Cry1a homologous protein sequences (Table S2) reveals extreme conservation in the N-terminal region (amino acids 1 to 505), while the remainder of the protein shows more variation. This is consistent with the position of the two important protein domains located within the first 500 amino acids, the DNA photolyase (amino acids 5–170) and the FAD binding domain (amino acids 213–486) (Fig. 2a, Table S3). The only cavefish-specific amino acid changes shown in the alignment are R > Q 263 and N > T 449. Both mutations are located in the FAD binding domain, but their impact on the protein activity should be moderately conservative (Grantham’s distances 43 and 85 respectively)^18^. Similarly, the alignment of Per2 homologous protein sequences showed only conservative or moderately-conservative amino acid substitutions in functional domains (Tables S2 and S3).

To analyze the selective constraint on *cry1a* and *per2*, we estimated the rate ratio ω of non-synonymous to synonymous substitutions using PAML^20^. The ratio of dN/dS (ω) is < 1 under purifying selection, approaches 1 under neutral rates of evolution, and is >1 under positive selection. As shown in Table S4, *cry1a* seems to evolve under purifying selection as expected for functional, conserved genes (ω = 0.064, Model B vs Model A, p < 0.05) and the rate of evolution of the cavefish lineage (ωPA = 0.060) is not significantly different from that of the rest of the tree (Model A vs Model C, p = 0.84). *per2* shows a very similar pattern, although with higher overall rate of evolution compared to *cry1a* (ω = 0.115, Model B vs Model A, p < 0.05). The branch model used to test if the cavefish lineage has a different rate of evolution compared to the rest of the tree was not supported by the data (ω = 0.115, ωPA = 0.114 Model A vs Model C, p = 0.93). The free ratio model, assuming that each branch in the phylogeny has an independent rate of evolution was favored for both genes over the one-ratio models (Model A vs Model D, p < 0.05).

Since gene expression results showed that the prematurely truncated *pa per2_tv3* is the most abundant transcript, we tested whether the expressed region of the gene showed a different pattern of evolution relative to the C-terminal truncated region. While the expressed protein sequence may still be under selective constraint, the truncated sequence is expected to behave as a neutral non-coding region, where the fate of mutations would be determined by random genetic drift. Contrary to this expectation, our results showed that both regions are conserved.

### Abnormal subcellular localisation of *P. andruzzii* Per2_tv3

**Figure 4:**
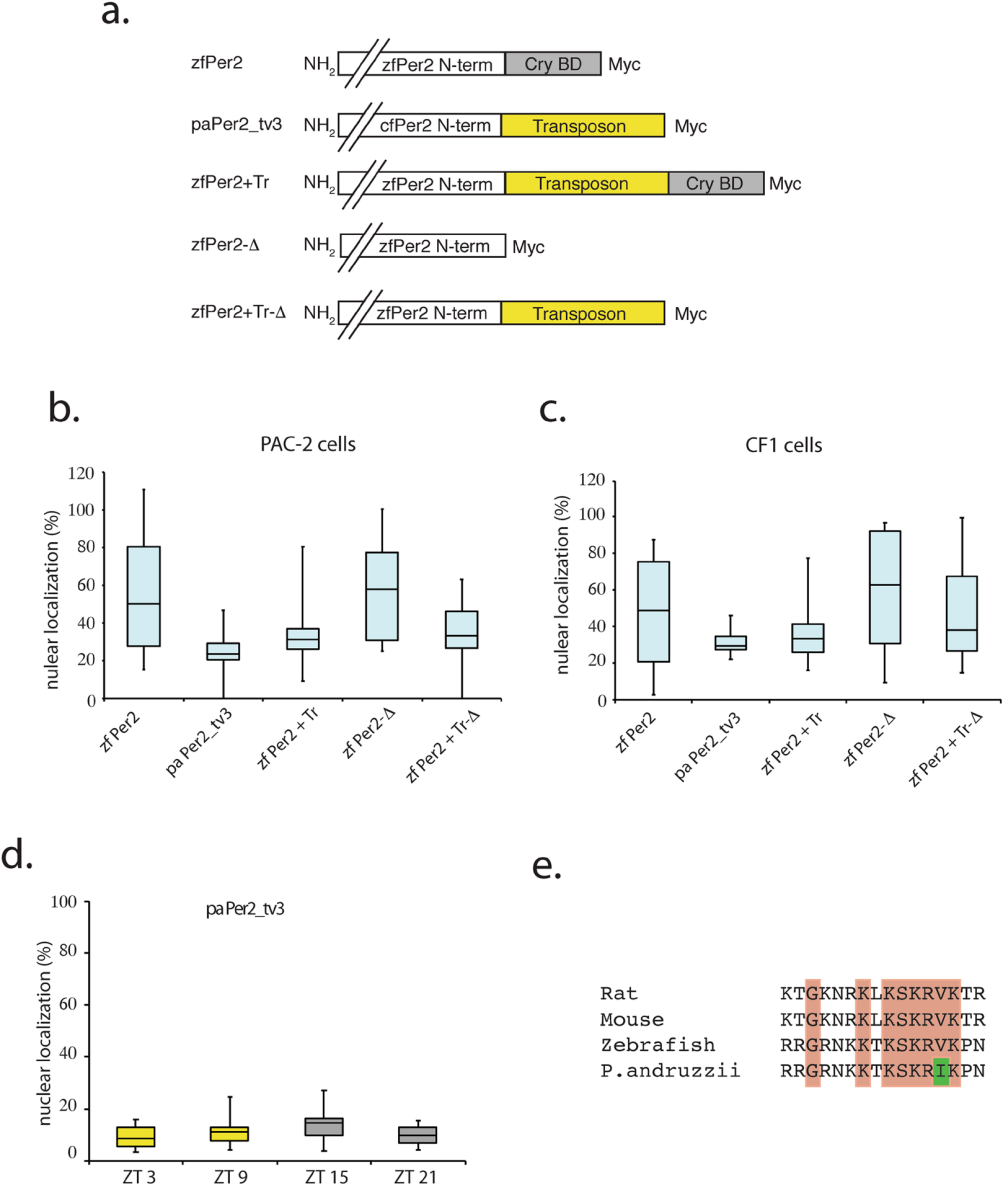
Subcellular localization of Per2 proteins in cavefish and zebrafish. (**a**) Schematic representation of Myc tagged expression constructs for the wild type zebrafish *per2* (zfPer2) and the more abundant cavefish (paPer2_tv3) form. Also, all the alternative engineered versions of zfPer2 mimicking the cavefish mutations are depicted. zfPer2+Tr incorporates the cavefish transposon intron-derived sequence, zfPer2-Δ carries a deletion of the Cry binding domain (Cry BD) and zfPer2+Tr-Δ incorporates both modifications present in the *P. andruzzii* Per2_tv3 protein. (**b**,**c**) Box-and-whisker plot diagrams representing the subcellular distribution of the various Per2 expression constructs transfected in (**b**) PAC-2 and (**c**) CF1 cells. Nuclear localization in % is plotted on the y-axes, while the names of the Per2 constructs are indicated on the x-axes. The whiskers (lines extending vertically from the boxes) represent the variability outside the upper and lower quartiles. The length of the boxes indicates the degree of dispersion in the data. The horizontal line within the boxes indicates the median. (**d**) Box-and-whisker plot diagrams representing the daily subcellular distribution of the cavefish pa Per2_tv3 construct transfected in zebrafish PAC-2 cells exposed to an LD cycle. Nuclear localization in % is plotted on the y-axis, while ZT times are indicated on the x-axis. All statistical analysis were performed by Kruskal-Wallis test followed by Dunn’s Multiple Comparison Test. (**e**) Amino acid sequence alignment of the bipartite nuclear localization sequence^34^ in the Per2 proteins of rat, mouse, zebrafish and *P. andruzzii*. Orange boxes highlight conserved amino acids while the green box represents the substituted amino acid in the cavefish protein with respect to the other 3 vertebrate species.

Previous studies in mammals have highlighted the importance of Cry and Per heterodimer formation as a prerequisite for nuclear translocation which in turn permits their down regulation of Clock- and Bmal-mediated transcription^22,23^. Given the insertion of a transposon sequence and lack of the Cry interaction domain at the C-terminus, does the more abundant Per2_tv3 isoform in *P. andruzzii* show a normal subcellular localization? We compared the subcellular localization of the zf Per2 and pa Per2_tv3 proteins using an immunofluorescence assay (Fig. 4). We transiently transfected each of the two Per2 expression constructs (Fig. 4a) into zebrafish (Fig. 4b) as well as cavefish cells (Fig. 4c). Comparing individual transfected cells revealed that the zf Per2 protein showed a variable distribution with both nuclear and cytoplasmic staining visible in each cell type (a mean of approximately 50% nuclear staining, Fig. 4b,c). However, in both cell types, pa Per2_tv3 was significantly enriched in the cytoplasm (approximately 20–30% nuclear staining, Fig. 4b,c) (Kruskal-Wallis test, p < 0.0001 for both cell lines). We next tested whether the insertion of the transposon sequence, the loss of the Cry interaction domain or, alternatively, the presence of additional single amino acid substitutions contributed to the cytoplasmic enrichment of the pa Per2_tv3 protein. We engineered the *zfper2* cDNA (zf Per2) to mimic the deletion of the Cry interaction domain (zfPer2-Δ), the insertion of the transposon-derived region present in pa Per2_tv3 (zf*per2*+Tr) or to carry both modifications (zf*per2*+Tr-Δ) (Fig. 4a). Transfection of these constructs into PAC-2 (Fig. 4b) and CF1 (Fig. 4c) cell lines followed by immunofluorescence analysis revealed a significant cytoplasmic enrichment of the constructs containing the transposon sequence relative to the constructs lacking this sequence which showed a similar nuclear and cytoplasmic distribution in both fish cell lines (Kruskal-Wallis test followed by Dunn’s Multiple Comparison Test, p < 0.0001 for both cell lines). Given that in other model systems Per2 protein function changes over the course of the circadian cycle^24^, we decided to test whether the cytoplasmic localization of the more abundant cavefish protein may be influenced by the time of day. Thus, we transfected the pa Per2_tv3 expression construct into zebrafish cells followed by exposure to a LD cycle. Immunofluorescence assayed at different time points throughout one 24 hours cycle showed no significant change in the subcellular localization of the pa Per2_tv3 form (Fig. 4d). Thus, together these results point to the insertion of the transposon-derived sequences rather than the loss of the Cry interaction domain as being the major determinant of the predominantly cytoplasmic subcellular localisation of the pa Per2_tv3 protein.

## Discussion

This work represents an important step forwards in our understanding of how evolution for millions of years in perpetual darkness can affect the light-entrainable circadian clock. We previously showed a general loss of light induced clock gene expression in the cavefish *P. andruzzii*. This lack of a gene expression response to light is due at least in part to the accumulation of loss of function mutations in key non-visual opsin photoreceptors^12,13^. Here we reveal the presence of additional mutations affecting a light-responsive, core circadian clock component, the *per2* gene. We have identified three splicing variants that encode a full-length Per2 protein as well as two C-terminally truncated proteins. Interestingly, of the three splicing variants, a form lacking the Cry interaction domain and carrying the insertion of a transposon-derived sequence at the C-terminus represents the more abundant isoform (pa *per2_tv3*). It is tempting to speculate that it is the particular pattern of post transcriptional processing of this transcript that renders it inherently more stable than the others. This might be linked to the presence of a premature termination codon (PTC) in exon 19 followed directly by an alternative 3′UTR sequence and polyadenylation site. The absence of Exon Junction Complexes formed by UPF proteins downstream of the PTC in this isoform combined with the alternative 3′UTR sequence may result in the transcript being a less efficient target for the nonsense-mediated decay pathway^25–27^. Alternatively, the incorporation of the transposon-derived sequences into this transcript may also favour its stability^28^.

The pa *per2_tv3* transcript variant encodes a predominantly cytoplasmic protein in contrast to the normal zfPer2 form which is distributed both in the cytoplasm and nucleus. Our results suggest that loss of the Cry interaction domain is not responsible for this shift in localization. Previous work using co-immunoprecipitation assays has documented physical interaction between Cry1 and Per2. Furthermore, crystal structure analysis and mutational experiments have localized a Cry interaction domain at the C-terminus of the Per2 protein in both mammals and zebrafish^22,23,29–31^. Although the results of many studies have supported the notion that the interaction between Cry and Per proteins plays a key role in their transport to the nucleus, the regulation of subcellular localization of the Per2 protein appears to be more complex. Some studies have described mPER2 as predominantly cytoplasmic in m*cry1*/m*cry2* deficient mutants and SCN cells^23,32^. Other reports place mPER2 protein as being distributed within the nucleus and cytoplasm, or even being exclusively nuclear in m*cry1*/m*cry2* double deficient mouse embryonic fibroblasts^30^ and in HER911 (human embryonic retinoblast) cells^24^. The mPER proteins have been reported to translocate from the cytoplasm to the nucleus upon co-transfection with mCRY1 in both NIH3T3 and COS7 cells^22,23^. However, zf Per2 transfected in NIH3T3 cells showed a cytoplasmic localization, and its distribution was not affected by co-expression with zf Cry^31^.

Outside of the cryptochrome interaction domain, amino acid sequences in the mPER2 protein serving as a cytoplasmic localization domain (CLD), nuclear localization signals (NLS) and nuclear export signals (NES) also appear to play a role in defining PER localization in the cell^33^. Indeed, deletions of the putative NLS in mPER2 have been reported to result in a predominantly cytoplasmic protein^30^. In this case, binding with CRY proteins might serve to stabilize the protein once it arrives in the nucleus and subsequently lead to its accumulation. Indeed, our results would tend to support this scenario. Our engineered version of zfPer2 lacking the Cry interaction domain (zfPer2-Δ) that mimics the cavefish variant pa Per2_tv2, was distributed both in the nucleus and cytoplasm as observed for the zebrafish Per2 protein (zfPer2), indicating that Per2 can enter the nucleus without requiring binding to Cry. However, in the case of zfPer2-Tr that mimics the transposon insertion present in pa Per2_tv3, the subcellular localization is predominantly cytoplasmic. These data imply that it is the insertion of the transposon-derived sequences that plays the principle role in conferring cytoplasmic localization. Nevertheless, it remains to be determined whether it is the transposon sequence itself or rather its particular location in the protein, for example shifting the position of the NLS relative to the C-terminus (see Fig. 2b), that is responsible for dictating the subcellular localization. While pa Per2_tv3 is a predominantly cytoplasmic protein (approximately 20–30% nuclear localization), zfPer2+Tr-Δ that mimics this cavefish isoform shows a higher nuclear localization (approximately 25–45%). Interestingly, within the highly conserved bipartite nuclear localization sequence located C-terminal to the PAS domains (see Fig. 2b and ref.^34^), the *P. andruzzii* protein exhibits an amino acid substitution relative to the zebrafish, mouse and rat Per2 proteins (Fig. 4e). It is tempting to speculate that this substitution may contribute to the differential nuclear localization of the zebrafish zfPer2+Tr-Δ protein, which contains the fully conserved NLS sequence.

A central unanswered question remains the functional consequences of mutations targeting light responsive clock genes in the cavefish. Addressing this question is particularly challenging given the multiple copies of clock genes resulting from genome duplication events that occurred early during teleost evolution. While in certain teleost species, two *per2* gene paralogs have been described^9^, our transcriptome and genome sequence analysis has failed to detect a second *per2* paralog in *P. andruzzii*, similar to the situation in zebrafish^9,13^. However, one key issue is how changes in the function of a single *per* gene can be compensated for by functional overlap with the other *per* gene orthologs. In part the result of the similarity between the *Drosophila* and vertebrate core clock mechanisms, both Cry and Per proteins are classically attributed to constituting the “negative” limb of the conserved core clock transcription - translation feedback loop. However, the situation seems to be less clear in the vertebrate clock mechanism. In mammals, Crys do appear to serve as the most potent repressors of Clock-Bmal driven transcription. Consistently, zf Cry1a functions as a potent repressor of zfClock-Bmal driven activation^35^. However, the role of Per proteins in regulating transcription appears to be more complex. Furthermore, while lack of Per2 protein function can clearly affect circadian rhythmicity in mammals and zebrafish, as well as in humans affecting the timing of sleep^6,36,37^, mPER2 has been implicated in numerous biological functions. This includes serving as a tumor suppressor, as well as playing a key role in immune system function and metabolism control^24^. In addition, in the case of the zebrafish core clock mechanism, while zf Per2 maintains zfClock-Bmal3 in the cytoplasm^31^ a dual role for Per2 has been suggested as it also enhances zf *bmal1b* expression^36^. In this regard it is tempting to speculate about the contribution the mutant Per2 isoforms might make to the blind, infradian circadian clock phenotype we have previously documented in the peripheral tissues of *P. andruzzii*^13^. Thus, aberrant Per2 protein function might contribute to the observed absence of rhythmic clock gene expression (*per1*, *clk1a*, *clk2*, and *cry1a*) in cavefish cells and tissues upon exposure to LD cycles. Changes in the negative elements of the core clock regulatory loop resulting from alterations in cytoplasmic levels of Per2 protein might also influence the overall timing of negative feedback within the core clock mechanism and thereby contribute to the abnormally extended period length of the cavefish clock cycle observed following synchronization by transient dexamethasone treatment^13^. In relation to all these questions, one important remaining issue is whether it is the cytoplasmic or nuclear tv_3 isoform protein that contributes to the cavefish phenotype.

Recently we have shown how relaxation of selective pressure on light responsive elements in *P. andruzzii*, specifically the melanopsin gene, contributes to the inability of peripheral clocks to respond to light^12^. Our discovery of mutations affecting the pa Per2 protein represents additional evidence for loss of elements of the circadian clock light input pathway during prolonged evolution in the absence of light. This is consistent with general theories attempting to explain troglomorphic phenotypes of cavefish evolution, where lack of adaptive significance of a given phenotype under constant darkness, such as the maintenance of functional eyes and visual photoreceptors, would be progressively lost due to the loss of visual sensing^38^. Additionally, the habitat shift from surface water to groundwater is often characterized by a reduction in effective population size that weakens the effect of natural selection. A recent study has shown how this population-size reduction is responsible for several genomic “relaxed” traits, such as the increase in genome size due to transposon insertions and a higher transcriptome-wide dN/dS in cave isopods^39^.

While selective constraints on the melanopsin gene were clearly relaxed, the whole *per2* coding region is conserved, even including the C-terminal region which is absent in the prevalent mRNA isoform and thus could be regarded as a pseudogenic sequence. This observation would suggest that the truncated pa Per2 protein, as well as the highly conserved Cry1a, play additional functional roles that are not exclusively linked with responsiveness to light and therefore may still be under selective pressure in the cave environment. Alternatively, one could speculate that the changes in pa Per2 are simply the first step of an ongoing relaxation of natural selection on light responsive pathways, and are not yet detectable in the coding sequence. Indeed, following the cave colonization and the associated change in selective constraint, mutations that render genes non-functional require a certain time to enter the population, depending on the population size and on the mutation rate. The integrity of certain genes may therefore persist for a long period purely by chance, unless selection favors degeneration. Subsequently, loss of function mutations might occur progressively. It is tempting to speculate that the loss of light-regulated *per2* expression may represent a first step in this process followed by the appearance of secondary mutations such as those described here which affect the subcellular localization of the protein and ultimately in combination with additional mutations, lead to complete pseudogenization. Interestingly, consistent with this scenario is the observation that the insertion of transposon sequences in genes is frequently encountered in cases of pseudogenization^40^.

## Electronic supplementary material

Supplementary Tables S1-S4
