# Supplementary material for: Mutations in blind cavefish target the light-regulated circadian clock gene, period 2
Source: Sci Rep. 2018 Jun 8;8:8754. doi: 10.1038/s41598-018-27080-2 (PMC5993827; doi:10.1038/s41598-018-27080-2)
Supplement: Supplementary file 1 — Supplementary Tables S1-S4 [file 41598_2018_27080_MOESM1_ESM.docx]

**Mutations in blind cavefish target the light-regulated circadian clock gene, *period 2*.**

Rosa Maria Ceinos^1,3^, Elena Frigato^2^, Cristina Pagano^1,3^, Nadine Froehlich^1^, Pietro Negrini^2^, Nicola Cavallari^2,3^, Daniela Vallone^1^, Silvia Fuselli^2^, Cristiano Bertolucci^2*^ and Nicholas S. Foulkes^1*^

^1^Institute of Toxicology and Genetics, Karlsruhe Institute of Technology, Eggenstein-Leopoldshafen, Germany.

^2^Department of Life Sciences and Biotechnology, University of Ferrara, Ferrara, Italy.

^3^Present addresses:

Rosa Maria Ceinos: Facultade de Bioloxía, Universidade de Vigo, Spain.

Nicola Cavallari: Institute of Science and Technology, Klosterneuburg, Austria.

Cristina Pagano: CNR, ISASI “E. Caianiello” Pozzuoli, Naples, Italy.

**Table S1**. List of primers used for PCR, RACE-PCR and qPCR.

1. List of primers used for qPCR data presented in Figure 1

| **Primer name** | **Primer sequence (5’->3’)** |
| --- | --- |
| qPCR *zf per2*_for  qPCR *zf per2*_rev | CTTCACCACACCATACAGG  GTCTGACGGGGACGAGTCT |
| qPCR *pa per2*_for  qPCR *pa per2*_rev | CCGCAAAGTTTCCTTCGTCA  CATTACTGCCCAGACTCCCA |
| qPCR *zf cry1a*_for  qPCR *zf cry1a* _rev | TCCGCTGTGTGTACATCCTC  CAAACACTGCAGCAAAAACC |
| qPCR *pa cry1a* _for  qPCR *pa cry1a* _rev | GGCTCCACGACAATCCTTCA  TGGGGAAGACATCGGTAGGT |
| qPCR zf/*pa act* _for  qPCR zf/*pa act* _rev | GTCTGGAGGTACCACCATGT ACATCTGCTGGAAGGTGGAC |
| qPCR *zf c-fos*_for  qPCR *zf c-fos*_rev | GCTCCATCTCAGTCCCAGAG  AGAGTGGGCTCCAGATCAGA |
| qPCR *pa c-fos*_for  qPCR *pa c-fos*_rev | GAATTCGCCAACCCTCCTCA  GTCAGTTCCATAGCCCTGCA |

1. List of primers used for PCR, RACE-PCR and qPCR data presented in Figure 3.

|  |  | **PCR product length** | | |
| --- | --- | --- | --- | --- |
| **Primer name** | **Primer sequence (5’->3’)** | **isoform1** | **isoform2** | **isoform3** |
| pa_per2ex16for  zf_per2ex18rev | CAGCTACAGCAGCACCATTGTACA GAGGATGGAGGCGGCCTGGTCTG | 682 bp | - | 912 bp |
| pa_per2ex17for  zf_per2ex19rev | TCTGGAGAGACAGAAAGGACA  GCGCATCGCTGTGTTGACCA | 998 bp | 75 bp | - |
| pa_per2ex18for  pa_per2ex19rev | TAACCCGCAGACTCCATTTC  ATGCATCGCTATGCTGACTG | 368 bp | - | - |
| pa_per2ex17Afor  zf_ per2ex20rev | AGGTTCTGGCAGCTCACACT  TTGGCCTTGTGGGATTTCTG | 1312 bp | 389 bp | - |
| pa_per2RACEex17for  pa_per2nst_ex17for | TGGGCCTGACCAAGCAGGTTCT  ATGCGTTCAAAGCCGACTGCTC |  |  |  |
| pa_per2RACEex20for  pa_per2nst_ex20for | CAACGGCTGTGGAACCTCTGCT  GGGTCTGCCAGCAGAACTGGAA |  |  |  |
|  |  |  |  |  |
| qPCR *per2*_for1  qPCR *per2*_rev1 | TGGAGAAGGCAGGAAGTGTT  GTCCTCTGGGAGAATGTCCA | 137 bp | - | - |
| qPCR *per2*_for2  qPCR *per2*_rev2 | CAGAACTGGAAGCAGCAACA  TTGGCCTTATGGGATTTCTG | 84 bp | 84 bp | - |
| qPCR *per2*_for3  qPCR *per2*_rev3 | GGTCTTCAATGAGCCCAAAA  GCCAGCATCAGTGTCTTGAA | - | - | 102 bp |

**Table S2** Accession numbers of the sequences used in molecular evolutionary analysis.

| **p*er2*** |  |  |  |  |  |  |  |  |  |  |  |  |  |
| --- | --- | --- | --- | --- | --- | --- | --- | --- | --- | --- | --- | --- | --- |
| >NM_182857.2:93-4292_Danio_rerio_period_circadian_clock_2_(per2)_mRNA | | | | | | | | |  |  |  |  |  |
| >XM_016255321.1:233-4444_PREDICTED:_Sinocyclocheilus_grahami_period_circadian_protein_homolog_2-like_(LOC107569272)_transcript_variant_X1_mRNA | | | | | | | | | | | | | |
| >XM_019074519.1:275-4435_PREDICTED:_Cyprinus_carpio_period_circadian_protein_homolog_2-like_(LOC109057293)_mRNA | | | | | | | | | | | | | |
| >XM_016548883.1:257-4393_PREDICTED:_Sinocyclocheilus_rhinocerous_period_circadian_protein_homolog_2-like_(LOC107737392)_mRNA | | | | | | | | | | | | | |
| >XM_016499507.1:261-4349_PREDICTED:_Sinocyclocheilus_anshuiensis_period_circadian_protein_homolog_2-like_(LOC107698490)_transcript_variant_X2_mRNA | | | | | | | | | | | | | |
| >XM_017718513.1:275-4441_PREDICTED:_Pygocentrus_nattereri_period_circadian_protein_homolog_2-like_(LOC108439864)_transcript_variant_X6_mRNA | | | | | | | | | | | | | |
| >XM_017495439.1:384-4502_PREDICTED:_Ictalurus_punctatus_period_circadian_protein_homolog_2-like_(LOC108280459)_transcript_variant_X5_mRNA | | | | | | | | | | | | | |
| >XM_018676636.1:364-4755_PREDICTED:_Lates_calcarifer_period_circadian_protein_homolog_2-like_(LOC108883459)_transcript_variant_X4_mRNA | | | | | | | | | | | | | |
| >XM_008294512.1:263-4624_PREDICTED:_Stegastes_partitus_period_circadian_protein_homolog_2-like_(LOC103366709)_transcript_variant_X8_mRNA | | | | | | | | | | | | | |
| >XM_010729980.2:265-4671_PREDICTED:_Larimichthys_crocea_period_circadian_protein_homolog_2-like_(LOC104918281)_transcript_variant_X4_mRNA | | | | | | | | | | | | | |
| >XM_019347598.1:1-4278_PREDICTED:_Oreochromis_niloticus_period_circadian_protein_homolog_2_(LOC100689707)_transcript_variant_X3_mRNA | | | | | | | | | | | | | |
| >XM_005912752.2:342-4619_PREDICTED:_Haplochromis_burtoni_period_circadian_protein_homolog_2-like_(LOC102311191)_transcript_variant_X3_mRNA | | | | | | | | | | | | | |
| >XM_017042402.1:259-4677_PREDICTED:_Cynoglossus_semilaevis_period_circadian_protein_homolog_2-like_(LOC103396076)_transcript_variant_X7_mRNA | | | | | | | | | | | | | |
| >XM_012916823.2:302-4498_PREDICTED:_Maylandia_zebra_period_circadian_protein_homolog_2-like_(LOC101485591)_transcript_variant_X3_mRNA | | | | | | | | | | | | | |
| >XM_017422543.1:306-4667_PREDICTED:_Kryptolebias_marmoratus_period_circadian_protein_homolog_2-like_(LOC108239667)_transcript_variant_X3_mRNA | | | | | | | | | | | | | |
| >XM_019878984.1:260-4693_PREDICTED:_Hippocampus_comes_period_circadian_protein_homolog_2-like_(LOC109521251)_transcript_variant_X3_mRNA | | | | | | | | | | | | | |
| >XM_013912435.1:302-4477_PREDICTED:_Pundamilia_nyererei_period_circadian_protein_homolog_2-like_(LOC102214448)_transcript_variant_X2_mRNA | | | | | | | | | | | | | |
| >XM_015956908.1:355-4599_PREDICTED:_Nothobranchius_furzeri_period_circadian_protein_homolog_2-like_(LOC107383961)_transcript_variant_X3_mRNA | | | | | | | | | | | | | |
| >XM_014031126.1:247-4626_PREDICTED:_Austrofundulus_limnaeus_period_circadian_protein_homolog_2-like_(LOC106534467)_transcript_variant_X2_mRNA | | | | | | | | | | | | | |

| ***cry1a*** |  |  |  |  |  |  |  |  |  |
| --- | --- | --- | --- | --- | --- | --- | --- | --- | --- |
| >Danio_rerio_ENSDARG00000045768 | | | |  |  |  |  |  |  |
| >gi\|558850318\|gb\|KF737846.1\| Astyanax mexicanus isolation-source Micos River cryptochrome 1a mRNA, complete cds | | | | | | | | | |
| >Clupea harengus_NW_012221194.1 | | | |  |  |  |  |  |  |
| >gb\|KP702272.1\|:838-2703 Siniperca chuatsi cryptochrome 1 (Cry1) mRNA, complete cds | | | | | | | | |  |
| >gi\|657548398:783-2660 PREDICTED: Stegastes partitus cryptochrome-1-like (LOC103356656), mRNA | | | | | | | | | |
| >gi\|808862710:532643-532800, 539598-539706, 540204-540346, 540442-540626, 540728-540816, 541036-541176, 541886-542197, 542331-542482, 542574-542776, 543307-543402, 543492-543587, 543691-543768, 543901-544001) Larimichthys crocea isolate SSNF unplaced genomic scaffold scaffold57, whole genome shotgun sequence | | | | | | | | | |
| >gi\|908519274:783-2675 PREDICTED: Oreochromis niloticus cryptochrome-1-like (LOC100694525), mRNA | | | | | | | | | |
| >gi\|583971024:752-2644 PREDICTED: Neolamprologus brichardi cryptochrome-1-like (LOC102777969), transcript variant X1, mRNA | | | | | | | | | |
| >gi\|548453565:780-2672 PREDICTED: Pundamilia nyererei cryptochrome circadian clock 1 (cry1), transcript variant X1, mRNA | | | | | | | | | |
| >gi\|939317897:760-2652 PREDICTED: Maylandia zebra cryptochrome-1-like (LOC101475237), transcript variant X1, mRNA | | | | | | | | | |
| >gi\|658917867:779-2650 PREDICTED: Poecilia reticulata cryptochrome-1-like (LOC103459142), mRNA | | | | | | | | | |
| >gi\|961795415:148-2025 PREDICTED: Poecilia mexicana cryptochrome-1-like (LOC106918704), mRNA | | | | | | | | | |
| >gi\|551496922:722-2599 PREDICTED: Xiphophorus maculatus cryptochrome-1-like (LOC102223115), mRNA | | | | | | | | | |
| >gi\|617410830:766-2643 PREDICTED: Poecilia formosa cryptochrome-1-like (LOC103140254), mRNA | | | | | | | | | |
| >gi\|961893201:776-2653 PREDICTED: Poecilia latipinna cryptochrome-1-like (LOC106955411), mRNA | | | | | | | | | |
| >gi\|671384926:3955-4112, 9415-9523, 9631-9773, 9875-10059, 10169-10257, 10403-10543, 10963-11274, 11424-11575, 11722-11924, 12091-12201, 12400-12498, 12587-12667, 12870-12970) Kryptolebias marmoratus cryptochrome 1 (Cry1) gene, complete cds  >gi\|974050874:736-2601 PREDICTED: Cyprinodon variegatus cryptochrome-1-like (LOC107085871), mRNA | | | | | | | | | |

| **Table S3** Distribution of amino acid changes (NS) in different protein domains of Per2 and Cry1a across the phylogeny analysed in this study (20 and 18 lineages respectively) and specific to the cavefish lineage.   \|  \| Total number of NS in the tree \| Private NS in cavefish \| Substitution \| Physicochemical effect * \| \| --- \| --- \| --- \| --- \| --- \| \| Per2 domains (AA interval) \|  \|  \|  \|  \| \| NLS (191-197) \| 6 \| 0 \|  \|  \| \| PASA (246-313) \| 10 \| 1 \| A>V 261 \| 64 \| \| PASB (386-452) \| 22 \| 2 \| T>A 393; P>S 404 \| 58; 74 \| \| PAC (459-502) \| 12 \| 0 \|  \|  \| \| NLS (877-891) \| 9 \| 0 \|  \|  \| \|  \|  \|  \|  \|  \| \| Cry1a domains (AA interval) \|  \|  \|  \|  \| \| DNA photolyase (5-170) \| 26 \| 0 \|  \|  \| \| FAD binding domain (213-486) \| 30 \| 2 \| R>Q 263; N>T 449 \| 54; 65 \| \|  \|  \|  \|  \|  \|  \|  \| \| *Grantham's distances: values ≥ 150 considered radical, 100 to 149 moderately radical, 50 - 99 moderately conservative, and < 50 conservative.  (Grantham 1974) \| \| \| \| \| \| \| \|  \|  \|  \|  \|  \|  \|  \| |
| --- | --- | --- | --- | --- | --- | --- | --- | --- | --- | --- | --- | --- | --- | --- | --- | --- | --- | --- | --- | --- | --- | --- | --- | --- | --- | --- | --- | --- | --- | --- | --- | --- | --- | --- | --- | --- | --- | --- | --- | --- | --- | --- | --- | --- | --- | --- | --- | --- | --- | --- | --- | --- | --- | --- | --- | --- | --- | --- | --- | --- | --- | --- | --- | --- | --- | --- | --- | --- | --- | --- | --- | --- | --- | --- | --- | --- |

**Table S4** Log Likelihood values (lnL), parameter estimates and Likelihood Ratio Tests of various models for the selective pressures acting on the *cry1a* and *per2* genes.

| **Gene: c*ry1a*** |  |  |  |  |  |  |  |  |  |
| --- | --- | --- | --- | --- | --- | --- | --- | --- | --- |
| Model | ω0 | ωPA | lnL^a^ | np^b^ | H0 | H1 | 2ΔlnL^c^ | df | *P*-value |
| A. One ratio: All branches have the same ω | 0,064 | ω0 | -9347,87 | 35 |  |  |  |  |  |
| B. One ratio: All branches have the same ω = 1 | 1 | ω0 | -10568,72 | 34 | B | A | 2441,70 | 1 | **0,0000** |
| C. Two ratio: ω0, ωPA | 0,0642 | 0,06022 | -9347,85 | 36 | A | C | 0,04 | 1 | 0,8415 |
| D. Free ratio: each branch has one ω |  | 0,06216 | -9322,49 | 67 | A | D | 50,76 | 32 | **0,0188** |
|  |  |  |  |  |  |  |  |  |  |
|  |  |  |  |  |  |  |  |  |  |
| **Gene: *per2 tv_1 (full length)*** |  |  |  |  |  |  |  |  |  |
|  | ω0 | ωPA | lnL^a^ | np^b^ | H0 | H1 | 2ΔlnL^c^ | df | *P*-value |
| A. One ratio: All branches have the same ω | 0,11548 | ω0 | -27916,72451 | 39 |  |  |  |  |  |
| B. One ratio: All branches have the same ω = 1 | 1 | ω0 | -30544,50294 | 38 | B | A | 5255,56 | 1 | **0,0000** |
| C. Two ratio: ω0, ωPA | 0,11553 | 0,1136 | -27916,7208 | 40 | A | C | 0,01 | 1 | 0,9314 |
| D. Free ratio: each branch has one ω |  | 0,11667 | -27809,93 | 75 | A | D | 213,59 | 36 | **0,0000** |
|  |  |  |  |  |  |  |  |  |  |
| ***per2 tv_3 (most abundant variant)*** |  |  |  |  |  |  |  |  |  |
|  | ω0 | ωPA | lnL^a^ | np^b^ | H0 | H1 | 2ΔlnL^c^ | df | *P*-value |
| A. One ratio: All branches have the same ω | 0,1121 | ω0 | -23552,68 | 39 |  |  |  |  |  |
| B. One ratio: All branches have the same ω = 1 | 1 | ω0 | -25845 | 38 | B | A | 4584,25 | 1 | **0,0000** |
| C. Two ratio: ω0, ωPA | 0,1120 | 0,11423 | -23552,68 | 40 | A | C | 0,01 | 1 | 0,9258 |
| D. Free ratio: each branch has one ω |  | 0,11581 | -23450,64 | 75 | A | D | 204,08 | 36 | **0,0000** |
|  |  |  |  |  |  |  |  |  |  |
| ***per2 C-terminal region (absent in tv_2 and tv_3)*** |  |  |  |  |  |  |  |  |  |
|  | ω0 | ωPA | lnL^a^ | np^b^ | H0 | H1 | 2ΔlnL^c^ | df | *P*-value |
| A. One ratio: All branches have the same ω | 0,12186 | ω0 | -4319,769964 | 39 |  |  |  |  |  |
| B. One ratio: All branches have the same ω = 1 | 1 | ω0 | -4673,430799 | 38 | B | A | 707,32 | 1 | **0,0000** |
| C. Two ratio: ω0, ωPA | 0,12201 | 0,11505 | -4319,764936 | 40 | A | C | 0,01 | 1 | 0,9201 |
| D. Free ratio: each branch has one ω |  | 0,13858 | -4288,91 | 75 | A | D | 61,73 | 36 | **0,0048** |
